# Angiotensin-(1–7) abrogates angiotensin II-induced proliferation, migration and inflammation in VSMCs through inactivation of ROS-mediated PI3K/Akt and MAPK/ERK signaling pathways

**DOI:** 10.1038/srep34621

**Published:** 2016-09-30

**Authors:** Feng Zhang, Xingsheng Ren, Mingxia Zhao, Bing Zhou, Ying Han

**Affiliations:** 1 Key Laboratory of Cardiovascular Disease and Molecular Intervention, Department of Physiology, Nanjing Medical University, Nanjing, Jiangsu 211166, China

## Abstract

The proliferation, migration and inflammation of vascular smooth muscle cells (VSMCs) contribute to the pathogenesis and progression of several cardiovascular diseases such as atherosclerosis and hypertension. Angiotensin (Ang)-(1–7) and Ang II are identified to be involved in regulating cardiovascular activity. The present study is designed to determine the interaction between Ang-(1–7) and Ang II on VSMCs proliferation, migration and inflammation as well as their underlying mechanisms. We found that Ang-(1–7) significantly suppressed the positive effects of Ang II on VSMCs proliferation, migration and inflammation, as well as on induction of the phosphorylation of Akt and ERK1/2 and increase of superoxide anion level and NAD(P)H oxidase activity in VSMCs, whereas Ang-(1–7) alone had no significant effects. This inhibitory effects of Ang-(1–7) were abolished by Mas receptor antagonist A-779. In addition, Ang II type 1 (AT_1_) receptor antagonist losartan, but not A-779, abolished Ang II induced VSMCs proliferation, migration and inflammation responses. Furthermore, superoxide anion scavenger N-acetyl-L-cysteine (NAC) or NAD(P)H oxidase inhibitor apocynin inhibited Ang II-induced activation of Akt and ERK1/2 signaling. These results indicate that Ang-(1–7) antagonizes the Ang II-induced VSMC proliferation, migration and inflammation through activation of Mas receptor and then suppression of ROS-dependent PI3K/Akt and MAPK/ERK signaling pathways.

Vascular smooth muscle cells (VSMCs) are an essential component of vascular walls and their highly differentiated contractile phenotype is critically responsible for maintaining vascular tone^1^. The proliferation and migration of VSMCs play crucial roles in atherosclerotic lesion formation and restenosis after angioplasty^2^. Accelerated proliferation of VSMCs is closely linked with hypertension^3^ and atherosclerosis^4^. The VSMC migration from media into intima is a critical event in the neointima formation in restenosis and is also an important feature in the formation of atherosclerotic plaques^5^. Vascular inflammation is considered to be involved in vascular remodeling in various cardiovascular diseases including hypertension and atherosclerosis^6^. Atherosclerotic plaques are complex lesions associated with chronic vascular inflammation, which contributes to VSMC proliferation and migration^7^.

Ang II is one of the biologically active peptides of the renin-angiotensin system (RAS). Ang II is pivotally involved in endothelial dysfunction, vascular inflammation and fibrosis in many cardiovascular diseases^8^,^9^,^10^,^11^. Most of the pathophysiologic actions of Ang II are largely mediated by AT_1_ receptors^12^. The Ang II/AT_1_R activity plays an important role in the proliferation and migration of VSMCs in the development of atherosclerosis^13^. Ang II/AT_1_ receptor is also demonstrated to be pro-inflammatory in the progression of atherosclerosis^14^. Ang II binding to the AT_1_ receptor results in stimulation of a variety of intracellular signaling pathways, including the phosphatidylinositol 3-kinase (PI3K)/Akt^15^ and the mitogen-activated protein kinases (MAPKs), contributing to the proliferation, migration and inflammation of VSMCs^16^,^17^. Prior studies have indicated that the diverse actions of Ang II in the vasculature are mediated by NAD(P)H oxidase derived reactive oxygen species (ROS), via activating multiple signaling molecules, including PI3K/Akt or MAPK^18^,^19^,^20^.

Angiotensin converting enzyme 2 (ACE2) was recently identified as a multifunctional monocarboxypeptidase responsible for the conversion of Ang II to Ang-(1–7)^21^. Ang-(1–7) is recognized to be an important therapy for vascular disorders associated with vascular remodeling that are likely mediated by specific Mas receptors and are selectively blocked by its specific antagonist D-Alanine-Ang-(1–7) (A-779)^22^,^23^. Ang-(1–7) is recently found to have a protective role in systemic hypertension, oxidative stress and tubulointerstitial fibrosis in diabetic mice^24^. The Ang-(1–7) levels were significantly decreased 2 weeks after vascular balloon injury, which was rescued by rosuvastatin treatment^25^. Ang-(1–7) abolishes advanced glycated end product (AGE) -induced cellular hypertrophy and myofibroblast transformation via inhibition of ERK1/2^26^. Oral formulation of Ang-(1–7) improves lipid metabolism and prevents high-fat diet-induced hepatic steatosis and inflammation in mice^27^. Ang-(1–7) prevents Ang II-induced fibrosis in cremaster microvessels^28^. However, little is known in regard to the interaction of Ang-(1–7) and Ang II in VSMC proliferation, migration and inflammation, which are critical events in the process of atherosclerosis. The present study was designed to explore whether Ang-(1–7) ameliorated the Ang II-induced proliferation, migration and inflammation in VSMCs and the exact cellular mechanisms.

## Results

### Ang-(1–7) inhibited Ang II-induced proliferation in VSMCs

**Figure 1:**
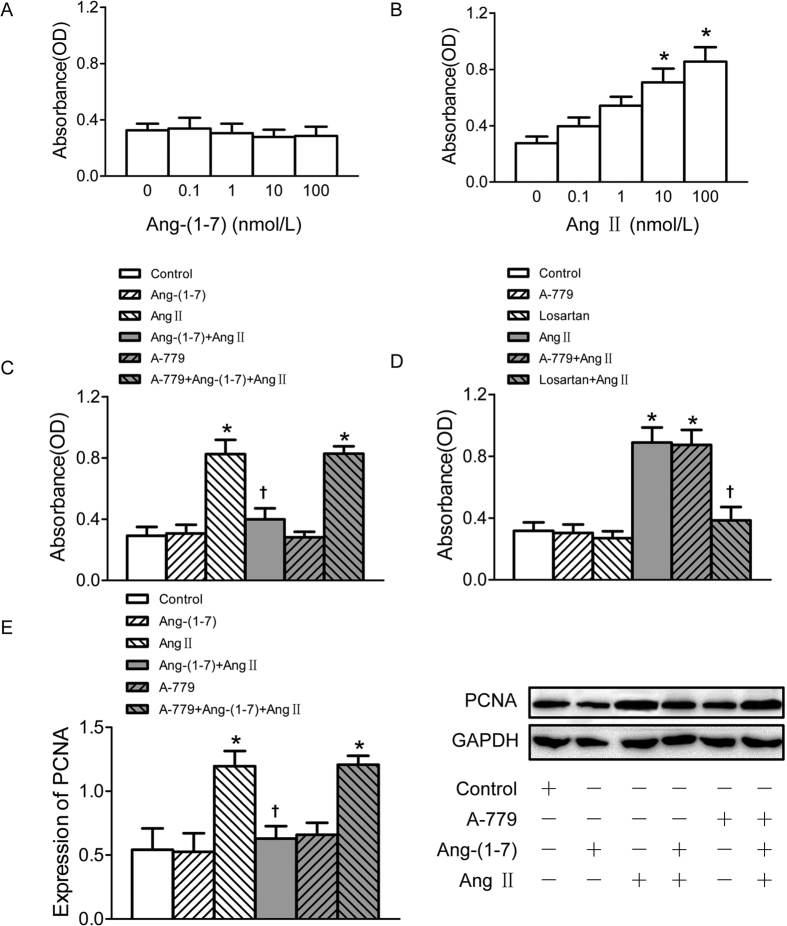
Effects on VSMCs proliferation. (**A**) Effects of 4 doses of Ang-(1–7) (0.1, 1, 10, 100 nmol/L) treatment on VSMCs proliferation; (**B**) effects of 4 doses of Ang II (0.1, 1, 10, 100 nmol/L) on VSMCs proliferation; (**C**) effects of control, Ang-(1–7) (100 nmol/L), Ang II (100 nmol/L), Ang-(1–7)+Ang II, A-779 (100 nmol/L), A-779+Ang-(1–7)+Ang II on VSMCs proliferation; (**D**) effects of A-779 (100 nmol/L) and losartan (100 nmol/L) on VSMCs proliferation or on Ang II (100 nmol/L) induced VSMCs proliferation response; (**E**) effects of control, Ang-(1–7) (100 nmol/L), Ang II (100 nmol/L), Ang-(1–7)+Ang II, A-779 (100 nmol/L), A-779+Ang-(1–7)+Ang II on PCNA expression in VSMCs. Values are mean ± SE. *P < 0.05 vs. control; ^†^P < 0.05 vs. Ang II. (**A–D**), n = 6 for each group; (**E**) n = 3 for each group.

The confluent VSMCs were seeded into 96 well-plates and incubated with different concentrations of Ang II and Ang-(1–7) separately for 24 h. All the pretreatments were administered 5 min before Ang II or Ang-(1–7) treatment. Cell Counting Kit-8 (CCK-8) assay showed that Ang-(1–7) treatment had no significant effect on the VSMC proliferation (Fig. 1A), but Ang II exerted an approximately 3-fold increase of VSMC proliferation at the dose of 100 nmol/L (Fig. 1B). Pretreatment of VSMCs with Ang-(1–7) significantly attenuated Ang II-induced VSMC proliferation, which was abolished by Mas receptor antagonist A-779 application before Ang-(1–7) (Fig. 1C). In addition, Ang II up-regulated proliferating cell nuclear antigen (PCNA) protein expression in VSMCs which were also obviously suppressed by pretreatment with Ang-(1–7), and this effect of Ang-(1–7) was inhibited by A-779 (Fig. 1E). Pre-incubation of AT_1_ receptor antagonist losartan, but not A–779 blocked the stimulatory effect of Ang II on VSMC proliferation. Neither losartan nor A-779 alone has effect to induce proliferation of VSMCs (Fig. 1D).

### Ang-(1–7) suppressed Ang II-induced migration in VSMCs

**Figure 2:**
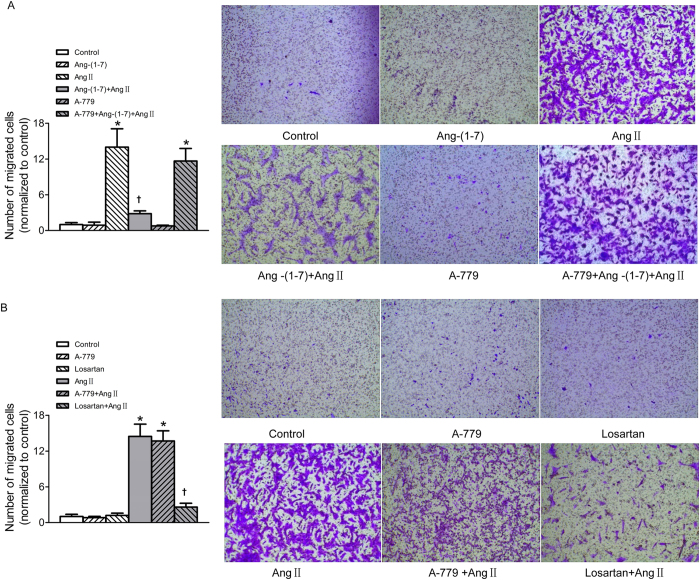
Effects on VSMCs migration. (**A**) Effects of control, Ang-(1–7) (100 nmol/L), Ang II (100 nmol/L), Ang-(1–7)+Ang II, A-779 (100 nmol/L), A-779+Ang-(1–7)+Ang II on VSMCs migration; (**B**) effects of A-779 (100 nmol/L) or losartan (100 nmol/L) on VSMCs migration and Ang II-induced VSMCs migration response. Values are mean ± SE. *P < 0.05 vs. control; ^†^P < 0.05 vs. Ang II. n = 3 for each group.

The transwell Boyden chamber experiment exhibited that treatment VSMCs with Ang II for 24 h markedly increased the number of VSMCs that migrated through transwell chamber. More important, after the VSMCs were pre-incubated with Ang-(1–7) for 5 min, Ang II-induced VSMC migration was significantly depressed. This inhibitory effect of Ang-(1–7) on Ang II was abolished by A-779 (Fig. 2A). AT_1_ receptor antagonist losartan effectively inhibited Ang II-induced VSMCs migration, but A-779 pretreatment was unable to prevent the VSMC migration induced by Ang II. Neither losartan nor A-779 alone has effect to induce migration of VSMCs (Fig. 2B).

### Ang-(1–7) retarded Ang II-induced inflammation in VSMCs

**Figure 3:**
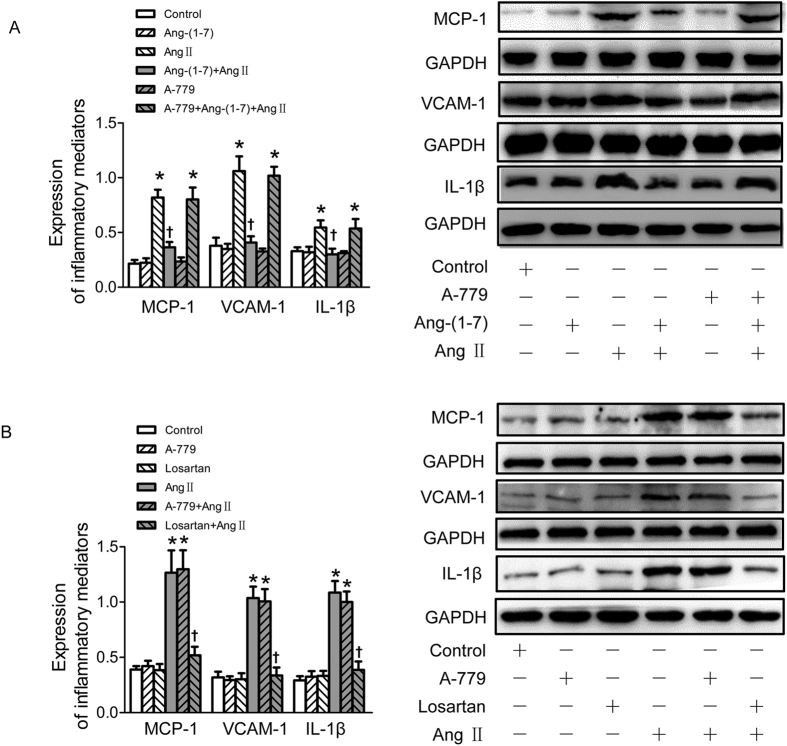
Effects on VSMCs inflammation. (**A**) Effects of control, Ang-(1–7) (100 nmol/L), Ang II (100 nmol/L), Ang-(1–7)+Ang II, A-779 (100 nmol/L), A-779+Ang-(1–7)+Ang II on MCP-1, VCAM-1 and IL-1β protein expressions in VSMCs; (**B**) effects of A-779 (100 nmol/L) or losartan (100 nmol/L) on the MCP, VCAM-1 and IL-1β protein expressions or Ang II (100 nmol/L) induced inflammation response in VSMCs. Values are mean ± SE. *P < 0.05 vs. control; ^†^P < 0.05 vs. Ang II. n = 3 for each group.

To examine whether Ang-(1–7) inhibited the inflammatory responses of VSMCs induced by Ang II, the expressions of inflammatory mediators of VSMCs including monocyte chemoattractant protein-1 (MCP-1), vascular cell adhesion molecule-1 (VCAM-1) and interleukin (IL)-1β were determined by Western Blot after Ang II application for 24 h. Pretreatment with Ang-(1–7) significantly retarded Ang II-induced inflammatory responses of VSMCs associated with up-regulated MCP-1, VCAM-1 and IL-1β expressions, and this effect of Ang-(1–7) was blocked by A-779 (Fig. 3A). As expected, pretreatment with losartan, rather than A-779 blocked Ang II-induced VSMC inflammation. Neither losartan nor A-779 alone has effect to induce inflammatory response of VSMCs (Fig. 3B).

### Ang-(1–7) prevented Ang II-induced phosphorylation of Akt and ERK1/2

**Figure 4:**
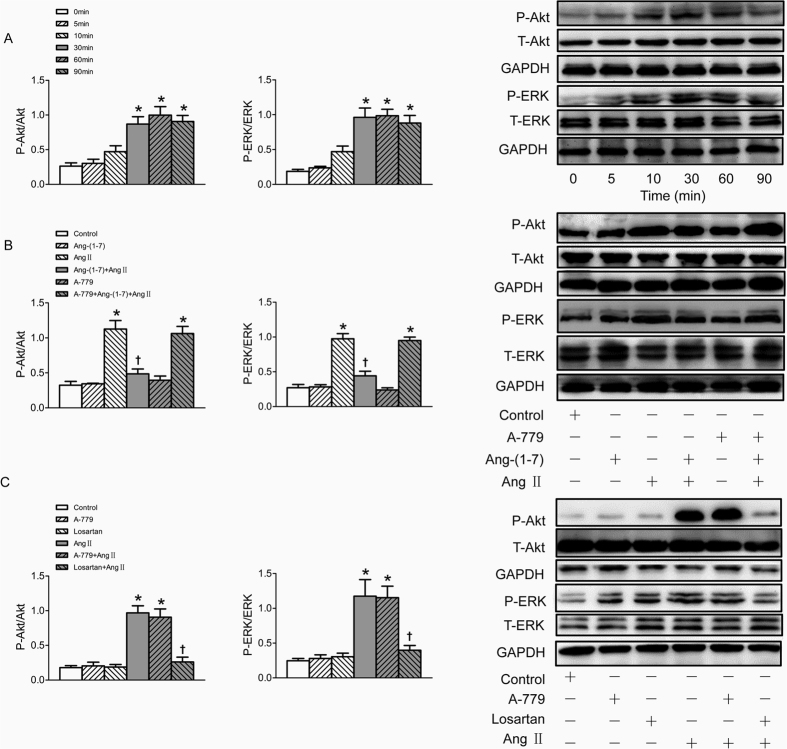
Effects on the phosphorylation of Akt and ERK1/2 in VSMCs. (**A**) time effects of Ang II (100 nmol/L) on the Akt and ERK1/2 phosphorylation; (**B**) effects of control, Ang-(1–7) (100 nmol/L), Ang II (100 nmol/L), Ang-(1–7)+Ang II, A-779 (100 nmol/L), A-779+Ang-(1–7)+Ang II on the Akt and ERK1/2 phosphorylation; (**C**) effects of A-779 (100 nmol/L) or losartan (100 nmol/L) on the Akt and ERK1/2 phosphorylation and Ang II induced phosphorylation response; Values are mean ± SE. *P < 0.05 VS. 0 min or control. ^†^P < 0.05 VS. Ang II. n = 3 for each group.

The phosphorylation of Akt and ERK1/2 were markedly increased after treatment VSMC with Ang II for 30 min, lasting at least 60 min. However, total ERK1/2 (t-ERK1/2) and total Akt (t-Akt) protein levels were not influenced by Ang II (Fig. 4A). Pretreatment VSMCs with Ang-(1–7) for 5 min significantly inhibited Akt and ERK1/2 phosphorylation induced by Ang II, and this effect was also blocked by A-779 (Fig. 4B). The increased phosphorylation of Akt and ERK1/2 induced by Ang II were effectively blocked by pre-incubation of losartan, rather than A-779, while either losartan or A-779 alone has no effect to induce phosphorylation of Akt and ERK1/2 in VSMCs (Fig. 4C).

### Ang-(1–7) diminished Ang II-induced ROS production

**Figure 5:**
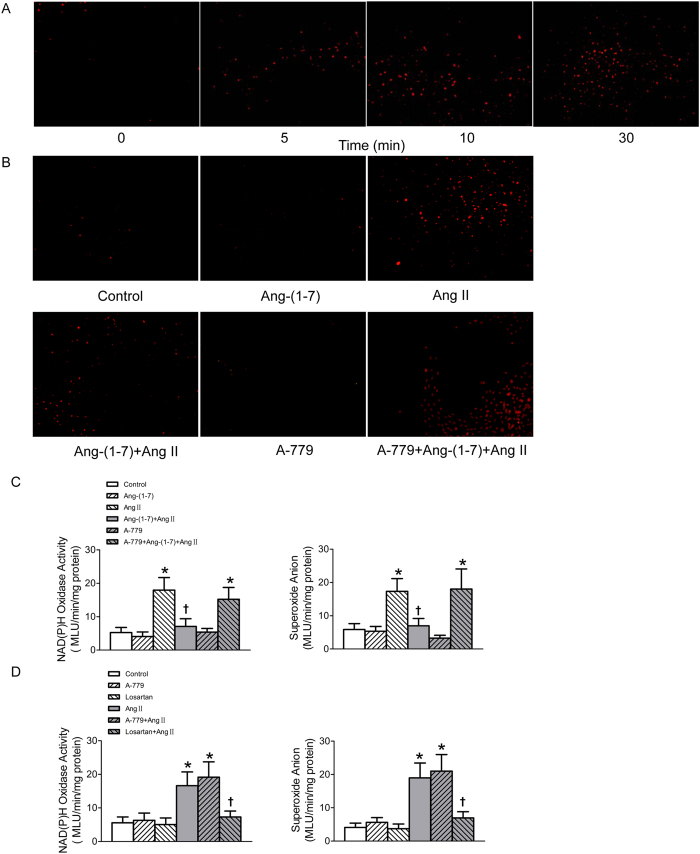
Effects on NAD(P)H oxidase activity and superoxide anions production in VSMCs. (**A**) Time effects of Ang II (100 nmol/L) on the superoxide anions production determined by DHE (10 μmol/L) probe; (**B**) effects of control, Ang-(1–7) (100 nmol/L), Ang II (100 nmol/L), Ang-(1–7)+Ang II, A-779 (100 nmol/L) or A-779+Ang-(1–7)+Ang II on the superoxide anions production in VSMCs determined by DHE probe; (**C**) effects of control, Ang-(1–7) (100 nmol/L), Ang II (100 nmol/L), Ang-(1–7)+Ang II, A-779 (100 nmol/L) or A-779+Ang-(1–7)+Ang II on the activity of NAD(P)H oxidase and superoxide anions production in VSMCs determined by lucigenin-derived chemiluminescence; (**D**) effects of losartan (100 nmol/L) or A-779 (100 nmol/L) on the activity of NAD(P)H oxidase and superoxide anions production in VSMCs as well as Ang II induced respones determined by lucigenin-derived chemiluminescence. Values are mean ± SE. *P < 0.05 VS. control. ^†^P < 0.05 VS. Ang II. n = 5 for each group.

The ROS production was detected after the confluent VSMCs were stimulated by Ang II (100 nmol/l) for different time (0, 5, 10, 30 min). Both dihydroethidium (DHE) fluorescence intensity and lucigenin-derived chemiluminescence method results showed that the superoxide anions production in VSMCs was remarkably enhanced upon Ang II stimulation for 30 min (Fig. 5A,C). NAD(P)H oxidase activity in VSMCs was also augmented in Ang II-treated VSMCs (Fig. 5C). The dramatic increases in superoxide anion level and NAD(P)H oxidase activity of VSMCs induced by Ang II were diminished by pre-treated with Ang-(1–7), and this effect of Ang-(1–7) was blocked by A-779 (Fig. 5B,C). The increased superoxide anion level and NAD(P)H oxidase activity in VSMCs induced by Ang II were blocked by pre-incubation of losartan, but not A-779. Both losartan and A-779 alone have no effect to influence superoxide anion level and NAD(P)H oxidase activity of VSMCs (Fig. 5D).

### NAD(P)H oxidase-derived superoxide anion activated the PI3K/Akt and MAPK/ERK signaling pathways in Ang II simulated VSMCs

**Figure 6:**
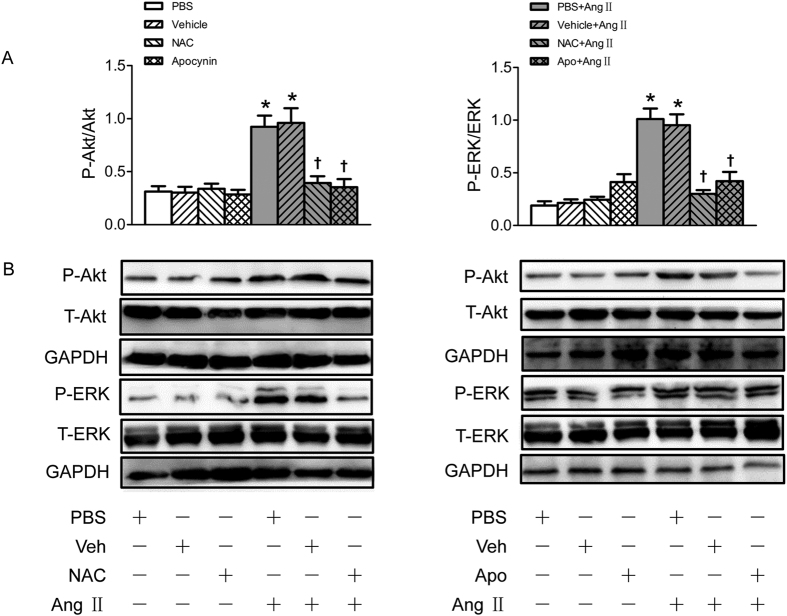
Effects of NAC and apocynin (Apo) on the phosphorylation of Akt and ERK1/2 in VSMCs. (**A**) Effects of NAC (1 mmol/L) and apocynin (Apo) (100 μmol/L) on the Akt and ERK1/2 phosphorylation and Ang II induced phosphorylation response in VSMCs; (**B**) Representative images of Western blot showing the effects of NAC and apocynin. Values are mean ± SE. *P < 0.05 VS. PBS or Vehicle. ^†^P < 0.05 VS. PBS+Ang II or Vehicle+Ang II. n = 3 for each group.

Pretreatment with either N-acetyl-L-cysteine (NAC) (a superoxide anions scavenger) or apocynin (a NAD(P)H oxidase inhibitor) abolished the effects of Ang II on the activation of Akt and ERK1/2 (Fig. 6).

## Discussion

Growing evidences suggest that dysfunction of VSMCs including proliferation, migration and inflammation is involved in the development of several diseases such as atherosclerosis and hypertension^13^. The Ang II/AT_1_ receptor activity plays an important role in the proliferation, migration and inflammation of VSMCs in the development of the above diseases^29^. The present study demonstrates new findings that Ang-(1–7)/Mas receptor activity significantly inhibited Ang II-induced VSMC proliferation, migration and inflammation through inactivation of superoxide anions-mediated PI3K/Akt and MAPK/ERK signaling pathways in VSMCs.

VSMCs are quiescent in healthy mature vascular tissue, however, they are activated and initiated abnormal proliferation and migration in response to vascular injury^30^. Vascular injury also causes vascular inflammation which combining with proliferation and migration are recently accepted to be as the key contributors in the pathophysiology of hypertension and atherosclerosis^10^. Inhibition of VSMC proliferation, migration and inflammation is an important strategy for therapy of atherosclerosis-related diseases^31^. Among various circulatory factors, Ang II is a well-characterized pathophysiological culprit peptide which has various actions for not only maintaining the blood volume, modulating blood pressure, but also promoting the VSMC proliferation, migration, inflammation and apoptosis via AT_1_ receptors in hypertension and atherosclerosis^32^,^33^. Incubation of rat tubular epithelial cells with Ang II significantly increased key pro-inflammatory cytokines expressions, which was blocked by losartan^34^. It is known that employment of Ang II receptor blockers may be helpful to abate inflammation processes and disease progression^35^. Inhibition of Ang II/AT_1_ activity has protective effects on VSMCs^36^,^37^. Ang-(1–7) is another major active component in the RAS, which can be converted from Ang II by ACE2^21^. Studies reported that Ang-(1–7) and Ang II have complicated interactions in different parts of the body in different animal models. Some studies have shown that Ang-(1–7) plays an opposite role to Ang II and inhibits the effects of Ang II in some peripheral tissues^38^,^39^. Ang-(1–7) prevents Ang II-induced fibrosis in cremaster microvessels^28^. The Ang-(1–7)/Mas receptor axis is counteractive to Ang II-induced proliferation, migration and inflammation in human brain VSMC and cerebral microvessels^40^,^41^. ACE2 deficiency accelerated Ang II-induced mRNA expressions of inflammatory cytokines, including MCP-1 and IL-1β^42^. In the present study, we found that Ang II drastically stimulated the VSMC proliferation and migration, and markedly up-regulated the expressions of inflammatory mediators including MCP-1, VCAM-1 and IL-1β, while Ang-(1–7) had no significant effects to induce VSMC proliferation, migration and inflammatory responses. However pretreatment with Ang-(1–7) on VSMCs significantly inhibited Ang II-induced proliferation, migration and inflammatory responses. These protective effects of Ang-(1–7) on VSMCs were blocked by Mas receptors inhibitor A-779. These results indicate that Ang-(1–7) is a potential antagonist in the disruption of Ang II induced excessive proliferation, migration and inflammation of VSMCs and these effects of Ang-(1–7) are done through activation of Mas receptors. We also found that blockade of AT_1_ receptors, but not Mas receptors, obviously diminished the proliferation, migration and inflammatory responses in VSMCs induced by Ang II, which indicates that the positive effects of Ang II on the VSMCs to induce proliferation, migration and inflammation is AT_1_ receptor dependent.

Oxidative stress is a critical modulator in the progression of hypertension and atherosclerotic lesions^35^. NAD(P)H oxidase is emerged as a multi-component enzyme complex and one of major origins of the superoxide anions in vascular system^43^. NAD(P)H oxidase-derived ROS play a critical role in Ang II-induced proliferation and migration of VSMCs^44^,^45^. Increased ROS is involved in the pathogenesis of Ang II-dependent hypertension^46^. Suppression of ROS by knockdown of NAD(P)H oxidase largely inhibited the Ang II-induced proliferation, inflammation in human mesangial cells^47^. We also previously revealed that the NAD(P)H oxidase-derived superoxide anions in the paraventricular nucleus (PVN) are responsible for Ang II-induced sympathetic outflow in hypertensive rats^48^. Ang-(1–7) is recently reported to counteract the Ang II-induced ROS over-production in cerebral endothelial cells^8^. The nonpeptide AVE0991, an agonist of the Mas receptor, significantly attenuated ROS production in Ang II-treated VSMCs^49^. Our results in the present study also showed that both NAD(P)H oxidase activity and superoxide anion level increased significantly in Ang II-treated VSMCs. Pretreatment with Ang-(1–7) inhibited Ang II induced increases in NAD(P)H oxidase activity and superoxide anion level in VSMCs. This effect of Ang-(1–7) was also abolished by Mas receptors inhibitor A-779. These results indicated that NAD(P)H oxidase derived ROS may be responsible for mediating the effects of Ang II to induce VSMCs proliferation, migration and inflammation. Ang-(1–7) served as an antioxidant to counteract Ang II-induced dysfunction in VSMCs by activation of Mas receptors.

Both MAPK pathway and PI3K/Akt pathway are essential intracellular signaling pathways involved in the VSMCs migration and proliferation during the processes of atherogenesis^50^. The stimulation of ROS-mediated MAPKs and PI3K/Akt signaling pathways play an important role in the human gastric cancer BGC-823 cells apoptosis^51^. The ROS-dependent phosphorylation of MAPK and PI3K/Akt act as key mediators in 6-hydroxydopamine-induced neuronal cell death^52^. In present study, we found that Ang II markedly increased the phosphorylation of Akt and ERK1/2 in VSMCs, which was blocked by losartan, but not A-779. Furthermore, pretreatment with either ROS scavenger or NAD(P)H oxidase inhibitor abolished the activation of Akt and ERK1/2 induced by Ang II. These results indicated that Ang II may increase NAD(P)H oxidase activity via AT_1_ receptors to induce superoxide anion over-production, then subsequent activation of PI3K/Akt and MAPK/ERK pathways, which is involved in the Ang II-elicited VSMCs proliferation, migration and inflammatory responses. More important, we found that the increased phosphorylation of Akt and ERK1/2 stimulated by Ang II were effectively inhibited by pre-incubation of Ang-(1–7) on VSMCs, which was blocked by Mas receptor inhibitor A-779. These results indicated that the activity of Ang-(1–7)/Mas receptor may have a potential role for vascular protection against Ang II effects via suppression of ROS-dependent PI3K/Akt and MAPK/ERK pathways.

In summary, the present study demonstrates that the ROS-dependent activation of PI3K/Akt and MAPK/ERK pathways may be critical contributors in mediating the Ang II-induced proliferation, migration and inflammation of VSMCs. Ang-(1–7) abrogates Ang II-induced proliferation, migration and inflammation by activation of Mas receptor in the VSMC membrane and then inactivation of ROS-mediated PI3K/Akt and MAPK/ERK signaling in cytoplasm. Application of antagonist of AT_1_ receptors or agonist of Mas receptors may provide beneficial strategies for cardiovascular diseases. Ang-(1–7) may be used as a therapeutic agent for hypertension and atherosclerosis.

## Methods

Experiments were carried out by using male Sprague–Dawley rats. Experiments and procedures were approved by the Experimental Animal Care and Use Committee of Nanjing Medical University and conformed to the Guide for the Care and Use of Laboratory Animal published by the US National Institutes of Health (NIH publication, 8th edition, 2011). The rats were housed in a temperature-controlled room with a 12 h–12 h light/dark cycle and with standard chow and tap water ad libitum.

### Culture of primary VSMCs

The primary culture of VSMCs from rats’ aortas was prepared as described previously^53^. Briefly, excised aortas were cut longitudinally and placed in digestion flasks with collagenase (type 1, 2 mg/ml; Sigma-Aldrich, St Louis, Missouri, USA) for 20 min at 37 °C in a shaker bath. Aortas were then cut into 1–2 mm segments, incubated with collagenase and elastase (0.5–1 mg/aorta; Sigma-Aldrich) in Hanks balanced salt solution for 1–2 h at 37 °C until single-cell suspension was achieved. VSMCs were maintained in Dulbecco’s modified Eagle’s medium (DMEM) supplemented with 10% FBS and 1% antibiotics (Gibco, MD, USA) at 37 °C in a 5% CO_2_ humidified incubator. The VSMCs were passaged at a ratio of 1:3 until confluence was reached. Cells in the second to sixth passages were used and cells at 80% to 90% confluence were arrested by incubating in serum-free DMEM for 24 hours before stimulation.

### Cell proliferation assays

The proliferation of VSMCs was evaluated with Cell Counting Kit-8 kits (CCK-8, Beyotime Institute of Biotechnology, Shanghai, China) as we previously reported^54^.

### Cell migration assays

The VSMC migration was assessed by a Boyden chamber assay. In brief, the quiescent VSMCs were seeded onto the upper surface of an Millicell transwell chamber of 8-μm (Merck Millipore, Billerica, Massachusetts, USA) in serum-free medium and then pretreated with chemicals for 5 minutes, then treated with the addition of Ang II only in the lower chamber. After that cells were incubated at 37 °C in air containing 5% CO_2_. After 24 h of incubation, the cells that migrated to the lower surface of the filter were fixed by methanol and stained by 1% crystal violet. The number of stained cells from at least 4 fields for each well was counted using a microscope^55^.

### Western Blot

The VSMCs were lysed in lysis buffer on ice for 15 min, and the soluble lysates were centrifuged at 4 °C for 10 min at 12,000 rpm. The protein concentration of supernatant in each sample was determined with BCA method. The equal amounts of proteins were subjected to sodium dodecyl sulfate-polyacrylamide gel electrophoresis (SDS-PAGE) by electrophoresis and transferred onto PVDF membranes. The membranes were blocked with 5% nonfat milk in Tris buffered saline with Tween-20 (TBST) for 60 min at room temperature and then hybridized overnight with indicated antibodies against PCNA (1:200, Santa Cruz Biotechnology, Santa Cruz, CA, USA), IL-1β (1:200, Santa Cruz Biotechnology, Santa Cruz, CA, USA), MCP-1 (1:200, Santa Cruz Biotechnology, Santa Cruz, CA, USA), VCAM-1 (1:200, Santa Cruz Biotechnology, Santa Cruz, CA, USA), phospho-ERK1/2 (P-ERK1/2) (Thr202/Tyr204) (1:1000, Cell Signaling Technology, Beverly, MA, USA), phospho-Akt (P-Akt) (Ser473) (1:1000, Cell Signaling Technology, Beverly, MA, USA), total-ERK (T-ERK) (1:1000, Cell Signaling Technology, Beverly, MA, USA) and total-Akt (T-Akt) (1:1000, Cell Signaling Technology, Beverly, MA, USA) at 4 °C overnight. After washing 3 times with TBST, the membranes were incubated with indicated secondary antibodies coupled to horseradish peroxidase for 1 h at room temperature. The signals of target proteins were visualized using the enhanced chemiluminescent reagent (Merck Millipore, Billerica, Massachusetts, USA). Densitometric analysis of bands was quantified with Image-J software (NIH, USA).

### Detection of intracellular superoxide anion level and NAD(P)H oxidase activity

After incubation, the VSMCs were washed with Hanks’ balanced salt solution (HBSS) and incubated with DHE (10 μM) for 30 min in a light-protected humidified chamber. The DHE fluorescence was measured at an excitation wavelength of 488 nm and an emission wavelength of 585 nm and the mean fluorescence intensity of DHE was assessed by Image-Pro Plus 6.0 by using the same parameters. The intracellular superoxide anion level and NAD(P)H oxidase activity were also measured with lucigenin-derived chemiluminescence method as we previously described^56^.

### Chemicals

Ang-(1–7) and A-779 were purchased from Bachem (Bubendorf, Switzerland). Losartan, NAD(P)H, apocynin, dimethyl sulfoxide (DMSO), lucigenin and Ang II were obtained from Sigma Chemical (St. Louis, MO, USA). NAC and DHE were obtained from Beyotime Biotechnology (Shanghai, China). Ang-(1–7) and Ang II were made fresh before each experiment.

### Statistical analysis

Comparisons between two groups were made by Student’s t test. One-way or two-way ANOVA followed by post hoc Bonferroni test was used when multiple comparisons were made. All data were expressed as mean ± SE. A value of P < 0.05 was considered statistically significant.

## Additional Information

**How to cite this article**: Zhang, F. *et al*. Angiotensin-(1–7) abrogates angiotensin II-induced proliferation, migration and inflammation in VSMCs through inactivation of ROS-mediated PI3K/Akt and MAPK/ERK signaling pathways. *Sci. Rep.* **6**, 34621; doi: 10.1038/srep34621 (2016).
